# Women oppressed in the daily lives and cultural practices of the akha people, Thailand: how can the situation change?

**DOI:** 10.1186/s40359-023-01361-6

**Published:** 2023-10-06

**Authors:** Pilasinee Wongnuch, Tawatchai Apidechkul, Peeradone Srichan, Thanatchaporn Mulikaburt, Siwarak Kitchanapaibul, Anusorn Udplong, Panupong Upala, Ratipark Tamornpark, Chalitar Chomchoei, Fartima Yeemard, Onnalin Singkhorn

**Affiliations:** 1 School of Health Sciences, Mae Fah Luang University, Chiang Rai, Thailand; 2 Center of Excellence for Hill Tribe Health Research, Mae Fah Luang University, Chiang Rai, Thailand; 3 School of Nursing, Mae Fah Luang University, Chiang Rai, Thailand

**Keywords:** Women oppressed, Inequity, Hill tribe, Akha, Norm, Culture

## Abstract

**Background:**

Gender equality is one of the most concerning issues globally. Females lacking equality could lead to several impacts, including health and economic impacts. Gender equality is often present in some minorities, such as the Akha hill tribe people who live in remote areas and have poor educational and economic statuses. This study aimed to understand the patterns and forms of women’s oppression through their norms and cultures.

**Methods:**

A qualitative method was used to elicit information from participants in twelve group discussions. The participants were Pha Mee and Ulau Akha people living in six selected villages along the border of Thailand and Myanmar. Twenty-two main questions were used as a guide in the discussions, which were grouped by gender and conducted by a same-gender moderator. The findings were extracted and formed according to a thematic approach.

**Results:**

A total of 72 Akha from six villages were invited to participate in the study: 29 males and 43 females. The average age was 47.7 years, 69.4% were married, 63.8% were Buddhist, 47.2% had never attended a school, and 47.2% worked in the agricultural sector. Several forms of Akha women’s oppression were identified: oppression through daily life, religious rituals, son preference, novels and cradle songs, naming ceremonies, and work performances. Many factors acted as unorthodox patterns to relieve the oppression of Akha women: religious conversion, educational impact, exposure to people from outside villages, and social and economic roles. Oppressed Akha women moved through four layers: individual, family, community, and external culture and modernization. The combination of culture and globalization was a key factor in gender inequity through these four layers to balance the pressures to oppress and resist.

**Conclusions:**

Akha women have lived under the power of men for several years, and these men have built up common features to control women in their society. Improving gender inequity is important for moving to a better stage of health, quality of life, and social roles, which will increase the power of all people to improve their society in the future.

**Supplementary Information:**

The online version contains supplementary material available at 10.1186/s40359-023-01361-6.

## Background

Under the current situation of human social progress, gender inequity is one of the most crucial issues. Capacity and contribution to any development aspect should not be dominated by a particular stage of gender. The United Nations (UN) set Sustainable Development Goal (SDG) No. 5, gender equality. This goal is to end all forms of discrimination to women and girls in any society [1]. Empowering women’s roles could improve women and children’s health and reduce individuals’ suffering, especially inequality, through cultural and norm patterns [2]. Suffering from the inequality of gender roles in society could impact several dimensions, such as economic growth [3], health [4], and access to public services [5]. Gender equality can be seen in several forms in various societies [6], including some specific minority populations [7] who live with poor educations and economic levels [8–10].

Thailand is a democratic country with a fundamental foundation that all citizens have the same rights to express their thoughts and which acts under a national constitution [11]. However, societal behaviors depend not only on laws and regulations but also on norms and cultures. The norms and cultures of some typical groups of people might overpower national laws and regulations and dominate people’s lives and behaviors. The mobilization of economics and education plays a key role in adjusting the balance of male-to-female roles in Thailand [12]. Many behaviors of one gender to dominate another are reducing [13]. However, the scenario remains common of females being oppressed in some minority populations, such as the hill tribe people living in northern Thailand.

The Akha are one of the six main groups of hill tribe people living in Thailand [14]. In 2021, approximately 4.5 million hill tribe people lived in Thailand, and Akha was ranked as the largest group. Akha has two majorities in Thailand: Pha Mee and Ulau. The two groups have some differences: language accent, clothing, and cultural practices. Some cultures and norms provide advantages to males, which might have been common in previous years or eras. However, under specific conditions and environments, including the new, widely accepted definition in society, some patterns of norms and cultures are defined as oppressing the female gender. Understanding the pattern and forms of gender equality through the social norms and cultures of the Akha people might eventually be used to promote the UN’s goal for gender equality. This study aimed to understand the patterns and forms of female oppression through the norms and cultures of the Akha people in northern Thailand.

## Methods

A qualitative method was used to gather information through twelve focus group discussions. The discussions elicited information regarding Akha cultures related to women’s roles and positions in their society. The focus group approach was used to gather information from the participants because the study objective was to understand the phenomena of being oppressed by a culture and its dynamics through the eras, and globalization was the most suitable approach [15, 16]. In addition, the discussion included women sharing their experiences of being oppressed by norms and socialization. Akha women and men aged 25 years and over living in six Akha villages from the Mae Fah Luang, Mae Chan, and Mae Sai districts, Chiang Rai Province, Thailand, were invited to participate in the study. All six villages are located along the border of Thailand and Myanmar and were the first Akha tribes who settled in Thailand from South China [14]. Two sub-major Akha groups live in Thailand: Pha-Mee and Ulau. These groups differ by accent and clothing [17]. However, some religious rituals and daily cultural practices are slightly different [18].

All six villages were identified as the original Akha villages in Thailand [19]: 221 households in the first village (Pha-Mee Akha), 72 households in the 2nd village (Pha-Mee Akha), 89 households in the 3rd village (Pha-Mee Akha), 177 households in the 4th village (Ulau Akha), 47 households in the 5th village (Ulau Akha), and 121 households in the last village (Ulau Akha).

Finally, a total of seventy-two (72) people participated in twelve focus group discussions divided into three categories: aged 35 years and below, 36–59 years, and 60 years and over. These three groups were classified based on their different beliefs, degrees of acceptance, and levels of adaptability throughout their lifespan and exposure to people in new modern society [20]. In addition to being Akha, they were born and lived in the selected Akha villages and were fluent in Thai. All participants were selected by a purposive method.

Group discussion questions were developed from the literature review and administered to 6 Akha people (two males and two females). The guideline was tested through a group discussion (five females and five males) in an Akha village in Mae Chang District, Chiang Rai Province, Thailand. Finally, twenty-two main questions were generated in four categories: general information, women’s oppression through Akha rituals and ceremonies, gender roles and norms, existing forms and patterns of oppression, and the dynamics of traditional cultures and norms under modernization.

The Akha villages were purposively selected based on their history. Access to the villages was granted by the district government officer. The village headmen were contacted and provided essential information regarding the study. The selection criteria for the participants were provided to the village headmen, who were asked to recruit people who met the criteria. A five-day advance appointment was made before commencing the group discussion. All participants were again provided the objective, procedure, and rights of the participants before signing informed consent forms. For those who could not speak Thai (but were able to speak and listen to Thai), the content was read to them before they signed the form.

The discussion was divided into a male and a female group with a same-gender moderator and notetaker to avoid gender domination. Moderators had backgrounds as medical anthropologists, social epidemiologists, behavioral scientists, and public health professionals who had vast experience performing studies using a qualitative method and were familiar with the hill tribe people living in these areas. Before the discussion began, all participants and the moderator provided information to make themselves known to each other. All participants were asked permission to record the whole discussion flow. During the discussion, the moderator kept the discussion on the study context and encouraged the participants to share their thoughts securely. The moderator also maintained a balance of opportunity so all participants could share and respect all ideas and thoughts throughout the whole discussion period. Each discussion lasted 90–120 min. All focus groups were carried out in August, 2021.

In the data analysis, all recorded data were transcribed into text by research assistants. Errors were checked before sending the transcripts back to the participants to validate the content. The content was coded in the form of a coding tree. Afterward, primary thematic forms were extracted by eleven researchers who had different backgrounds (two medical anthropologists, two medical sociologists, two public health professionals, one psychologist, one behavioral scientist, one social epidemiologist, and two public health epidemiologists). The codes were transferred into the NVivo program (NVivo, qualitative data analysis software; QSR International Pty Ltd., version 11, 2015) for further analysis. Finally, the obtained information was structured and the findings were formed by the researchers. Before the final interpretation, the findings were returned to the participants for confirmation.

## Results

### General characteristics

A total of 72 Akha from six villages was invited to participate in the study: 29 males and 43 females. The average age was 47.7 years, 69.4% were married, 75.0% were animist or members of a traditional religion, 47.2% had never attended a school, 47.2% worked in the agricultural sector, and the median monthly income was 3,000 baht ($86) (Table 1).

**Table 1:** General characteristics of participants

Characteristics	n	%
Total	72	100.0
Akha		
Ulau	46	63.8
Pha-Mee	26	36.2
**Sex**		
Male	29	40.3
Female	43	59.7
**Age** (years) Min = 24, Max = 88, Mean = 47.79		
**Marital status**		
Married	50	69.4
Ever married	14	19.4
Single	8	11.2
**Religion**		
Animist or Traditional religion	54	75.0
Christian	18	25.0
**Education**		
No education	34	47.2
Primary school	9	12.5
High school	22	30.5
Diploma	5	7.0
University	2	2.8
**Occupation**		
Agriculture	34	47.2
Temporal employee	19	26.4
Unemployed	14	19.4
Merchant	3	4.2
Government officer	2	2.8
**Thai ID card**		
Yes	68	94.4
No	4	5.6

**Monthly income** (bath) Min = 500, Max = 28,000, Median = 3,000, IQR = 3,500

**Family members** (person) Min = 1, Max = 12, Mean = 4.94

**Number of children** (person) Min = 0, Max = 10, Mean = 2.75

The extracted study information was presented in terms of Akha women’s oppression forms, unorthodox patterns, and the current movement for new norms against Akha women’s oppression.

### Forms of oppression among akha women

Akha women in Thailand experienced at least six forms of oppression, detected among people in different age categories.

#### daily life oppression

After Akha women wed, they have no choice to return to their family of origin. They become their husbands’ assets immediately after marriage. When conflicts occur between Akha husbands and wives, women have no flight power, which is a cultural state that robs women of an important role against their husbands. A married woman may not return to her family of origin, because Akha believe that if she does, her parents will get sick. Moreover, in Akha culture, women must manage all the housework and eat after all other family members. In the family, Akha women must follow the instructions of their mothers-in-law unconditionally.

A 59-year-old Ulau Akha man [P#38]:

Akha women can choose their partners themselves. However, in some families, the women are chosen by their mothers-in-law. In doing so, a man’s family always looks for a good woman for their son. A good woman in Akha does good work in housework and farming and dresses well everyday, meaning she has the handicraft skills for traditional clothmaking.

A 76-year-old Ulau Akha woman [P#12]:

Every morning early, I am warned at approximately 3 a.m. by my father-in-law to wake up and work. I have to cook for everyone in the family and eat my own breakfast after they do. Then, I walk to the farm with my two kids and care for my children while I farm.

A 64-year-old Ulau Akha woman [P#17]:

In the past, we had a large family with 5–6 children in the house who stayed all together. All daughters-in-law were assigned to work different jobs in the morning and the whole day, such as pounding rice, fluffing cotton, feeding pigs, and carrying water. Meanwhile, men worked guarding the rice bucket. If a family had many sons, they had many daughters-in-law and children. A boy or girl knows his or her life roles that the adults perform every day.

A 77-year-old Ulau Akha man [P#29]:

My mother-in-law was the banker in the family who kept all the money. She had the highest authority to approve the use of money and to control and manage all spending in the family. All income from anyone in the family was required to be kept by the mother-in-law in a large pocket. If anyone needed to use money, he or she had to receive approval from my mother-in-law. If a family had a large amount of money, they kept it inside jar and put it inside land near their house. When a father-in-law died, the money was allocated only to the sons.

A 65-year-old Akha Phamee man [P#26]:

In Akha culture, a husband can hit his wife, but his wife cannot respond. If a wife responds to or hits her husband intentionally, her husband will eventually develop a serious problem and might die. When I was young, I saw a woman throw a plastic chair at her husband to hurt him, and it hurt his leg a little bit. Her husband died two months later. Then, whenever there was a conflict in the family, it was taken seriously in Akha culture; the village headman was asked to decide whether to remove a woman from her husband’s family.

Akha women were oppressed in their daily lives by people in their families and communities. Some patterns did not allow them to even have a choice, especially in the aspect of being an asset to her husband after getting married.

#### Religious ritual oppression

In Akha culture, no women are allowed to join in all religious practices. Many religious rituals or practices are conducted yearly, and only on Low Chin Cha is a girl or woman allowed to participate. On this day, women are expected to dress beautifully in traditional dresses. More than 10 other practices forbid women. The joining in many religious rituals was strongly restricted for women. A bad event will happen to a person or a family, including a community, if women are allowed to join.

A 41-year-old Akha Phamee man [P#43]:

In Akha culture, only a man can participate in our religious practices such as killing pigs or chickens for the rituals. If a family has only women, they cannot participate in the religious rituals anymore.

A 66-year-old Ulau Akha man [P#2]:

“In the Akha funeral ceremony, only a man can participate, which requires that they recite their previous generational names such as Bo-Che, Che-Sa, Sa-Cher, Cher-Gue, Gue-Cher, Cher-Choo, Choo-Yang, Yang-Lae, Lae-Chean, and Chean-Gor. Speaking the previous generational names in the funeral ceremony causes the dead to go to the peak place after death, and only a man can do that.

Almost none of the religious rituals of the Akha people allowed women to participate. This situation reflected male dominance among the Akha people.

#### Oppression through the son preference

Akha culture prefers sons to daughters. They believe that a son is the next footprint for the parents. A son’s name follows his father’s name, while a girl’s name is ignored. A son will handle all family assets in the next generation. The Akha people believe that five daughters are not better than a son. A family who has a son has been respected by their previous generations and is respected by other people in the village. Moreover, a family that has a son has a better life.

A 29-year-old Phamee Akha woman [P#58]:

All Akha families need a son to be a descendant and manage the religious rituals in their family. Moreover, the Akha people believe that a son will be the representative of the father for the next generation.

An 81-year-old Phamee Akha man [P#25]:

A family must have at least one son. Without a son, the ritual cannot be performed. If the father passes away, only a son can perform all the religious practices.

A 33-year-old Ulau Akha man [P#73]”.

Men in Akha are superior and grandiose. If a family does not have a son, the family will not be able to continue their generations. The son is the descendant who guarantees life after the death of all family members.

A 39-year-old Phamee Akha man [P#42]:

In a family that does not have a child or son, the wife must accept that her husband will have a second or third wife until they obtain a son. The Akha people accept polygamy to have a son in the family. Having many wives in a family is common, and all sons from second wives will automatically be sons of first wives.

A 47-year-old Phamee Akha woman [P#54]:

If a family does not have a son, the death could not be placed the same as other deaths in families who had sons. The Akha people believe that a person who has no son will go to other, different places than those who have sons.

A 44-year-old Phamee Akha woman [P#57]:

Like this old lady: she has five daughters, but no son. She has no right to participate in the village religious rituals anymore. We believe that she will have trouble after death as well.

Male dominance was present through many beliefs of the Akha people. Having a son in a family was a basic necessity for maintaining the next generations, while a daughter was an asset to her husband.

#### Oppression through novels and cradle songs

Several Akha novels describe a boy’s performance rather than a girl’s. A boy or a man is always a hero in Akha stories. Boys are given the main role of maintaining their generations. Most dogmas have been formed and developed in Akha by men. The details of many cradle songs were formed by the male roles and preferred these to the female roles.

An 88-year-old Ulau Akha woman [P#16]:

The contents of Akha’s lullaby songs for sons and daughters are different. A song for sons teaches them how to practice as men while growing up, especially hunting wild animals. Meanwhile, a song for daughters teaches them how to handle their lives as good wives.

Among Akha society, stories and novels prefer boys and oppress girls. This practice indirectly retains female oppression through stories in Akha society.

#### Oppression through naming ceremonies

A boy’s name always reflects the family’s hope; boys are carefully named. A boy’s name is also linked to his father or grandfather’s name, especially when either of them was famous in their community. Male birthing and naming ceremonies are more elaborate than female ones. If a family has a boy, he is given the top of a leaf, which acts like a crown. Girls are not permitted this practice.

A 66-year-old Ulau Akha woman [P#2]:

In the birthing ceremony, girls and boys have different practices, including naming ceremonies, which are done by the father. In Akha culture, naming a boy is done much more carefully and always following the father’s name. For example, if the father’s name is Chean-Gor, the child will be named Gor-Ngo, Gor-Cher, or Gor-Per. A son’s name follows the last name of his father, while a girl’s name is not taken seriously among Akha people.

A 69-year-old Ulau Akha woman [P#14]:

At a birthing ceremony, a chicken is killed and three feathers are plucked to be pinned on the father’s hat. For a boy, the father pins up the feathers. If it is a girl, the feathers are pinned down. When the ceremony is finished, he puts the feathers in the eaves of the house. When a child is 13 days old, the mother carries her child out to the sacred village gate. If it is a girl, simply pick up three leaves from any kind of tree. If it is a boy, the mother needs three leaves from the tree’s top to boil and bath her son.

Naming ceremonies for boys were always more complicated and longer lasting than those for girls. This practice was another reflection of the Akha people favoring sons over daughters and oppressing women in Akha society.

#### Oppression through work performance

A woman, before getting married, will be assessed as suitable for a man by a look at her farming performance. If a woman has no weeds on her farm, she is assumed to have good performance. She would be a good marital choice for a man.

A 76-year-old Ulau Akha woman [P#12]:

A woman has to work on a farm, even if we are pregnant and close to giving birth. We have to bring a cutter to cut the umbilical cord in case we deliver at the farm. We have to do it by ourselves. I gave birth at my farm. On that day, I worked alone and gave birth alone. I cut my baby’s umbilical cord by myself and walked back home.

An 81-year-old Phamee Akha woman [P#23]:

Women have to work hard while men have much more freedom in their lives. We, as women, have to work a farm and plant flowers with a beautiful landscape on the mountain. We compete with other women with our farming products. A woman who is a good and productive farmer will receive gratitude from the villagers.

A 64-year-old Ulau Akha woman [P#17]:

When we return from farming, we have to carry a basket full of bamboo shoots. If you walk back with an empty basket, you will be called “Da-Kho”, meaning you are a sluggard.

A 73-year-old Ulau Akha men [P#28]:

A woman is not allowed to perform men’s duties such as judging a village problem. Any woman who joins the forum will be called “Nang-Ngue”, which is an unpleasant woman. This is very serious.

These statements reflect the oppression of women in Akha society. Women were expected to hard work and work is used as a tool to identify a good wife for a man.

### BUnorthodox patterns relieving the oppression of women

Several patterns were detected in adapting to current life among the Akha people, especially among women who relieved their oppression in Akha society. The relief was presented through many patterns or determinants.

#### Religious conversion

Some Akha families could not bear their daughters’ suffering, and they converted to Christianity. After many believers in Christianity began not favoring boys over girls, many Akha people became Christians. The speed of religious conversion was rapid in communities that had strict traditional religions, especially heavy male dominance.

A 42-year-old Phamee Akha woman [P#56]:

In the past, we wanted to get a son to perform Akha traditional ritual, but now we have converted to Christianity and Buddhism. There is no need for a son to perform the traditional ritual.

A 49-year-old Ulau Akha woman [P#8]:

We thought a lot about the difficulty of our traditional religious practices, and some of us did not have a son. Then, we decided to convert to Buddhist or Christianity. Today, we are very happy, and we do not need to worry about the practices.

A 60-year-old Phamee Akha woman [P#20]:

If we do not have a son, we cannot perform Akha traditional rituals. We can convert to Christianity. As Christian, we can also have a good life after death.

Religious conversion to Christianity was a major adaptation to a new norm and was believed to relieve suffering among the Akha people. This was one great option for the Akha people to not pass their suffering on to the next generation.

#### Educational impacts

Education was a significant contributor to reducing female oppression in Akha society. Girls who were educated had more successful lives and were relieved from female oppression in Akha society. Access to the educational system in Thailand is not restricted by gender; neither are most jobs in Thailand, and anyone can attain success.

A 31-year-old Phamee Akha man [P#44]:

Today, many Akha children are not at home. They go to school outside our villages. Many of them are not interested in inheriting traditional ancestral rituals. Then, it becomes difficult to follow our traditional religious practices.

A 43-year-old Ulau Akha man [P#33]:

In the past, we preferred to support our first son in his education over the other children. However, today, Akha families support the schooling of all their children, regardless of whether they are girls or boys to develop their interests. Once they graduate, they will not return to our village. They prefer to get jobs and have families in the city.

A 34-year-old Phamee Akha woman [P#50]:

Today, the hill tribe children and the children in the city are all the same. Schoolchildren are taught to do good things, such as not killing animals, including chickens, and pigs. Then, new generations do not accept the killing of animals for ritual traditions.

Education was an important tool for relieving female oppression in Akha society. Allowing everyone to have a proper education could improve individual capacity and job opportunities. Female oppression decreases when women are educated and employed. Young Akha women indicated that having their incomes could improve their purchasing power and reduce oppression from their husbands.

#### Exposure to people outside the village

In the context of globalization, the Akha people have been exposed to cultures and norms from people outside their village. Thus, the Akha people have combined their traditional norms and cultures with modern styles, especially in gender inequity. As a result, the perception of women’s oppression has women declined. Exposure to people outside the village was found in most of the younger age groups. The most important pattern was the combination of Akha and Thai cultures, which resulted in a decline in the stress of the oppressed women in Akha.

A 34-year-old Phamee Akha woman [P#53]:

“In previous days, we felt uncomfortable that women did not work all the time. Today, both men and women have the same responsibilities to work on farms and at home. Meanwhile, the relationship between mothers- and daughters-in-law has changed; it is not as strict as it was previously.”

A 33-year-old Ulau Akha woman [P#61]:

Today, if there is a village meeting, women must join; otherwise, it cannot come to a conclusion. Sometimes, women play a more important role than men. Women can be community leaders. In our village, our headman’s assistant is a woman.

A 33-year-old Ulau Akha woman [P4]:

Sometimes, we do not know if a person is Akha because we dress like urban people. Many of the Akha people do not dress in our traditional form.

A 31-year-old Phamee Akha woman [P51]:

We have never used Akha songs to make a child sleepy. We tell stories like the ones that school tells them. Our songs tell our kids to grow up healthy and get a job.

A 29-year-old Ulau Akha woman [P#69]:

Divorce is no longer a problem for Akha women. They can live alone, and there is no need to remarry like people believed in previous days. We have 40-year-old women in the village who are not getting married.

The oppression of Akha women was relieved because they were exposed to people outside their village. Exposure to Thai people who had much more gender equity and other mixed cultures impacted the decline in Akha women’s oppression.

#### Social and economic roles

As the new generations of the Akha people interact socially through different modes or approaches, including online platforms, they have the opportunity to meet and interact with people globally. Social interactions with other Akha people led them to gain and know related equity information and culture, especially in expressing their thoughts. Moreover, the individual and family economic status caused them to have greater power to negotiate with people in their families.

A 31-year-old Phamee Akha woman [P51]:

Everyone has a cell phone; we use LINE and Facebook. We do not need face-to-face interaction.

A 41-year-old Phamee Akha man [P43]:

Now, some Akha women can drive to the farm or go down to the city. They do not have to rely on men like in previous days. A woman can operate all trades by herself as we see on television. Akha women can do the same things as other women do.

A 29-year-old Phamee Akha woman [P58]:

Currently, women can trade like men. She can keep her money, with no need to give her money to her husband like in previous days. We also have smaller families compared to the previous generation.

Akha women had more power to relieve their cultural oppression when they interacted socially with people outside their villages through different media platforms or face-to-face interactions. Economics enhanced their negotiation power with people in their families and communities, helping them relieve cultural oppression.

### Dimensions of the shift to the new norms for oppressed Akha women

**Fig. 1:**
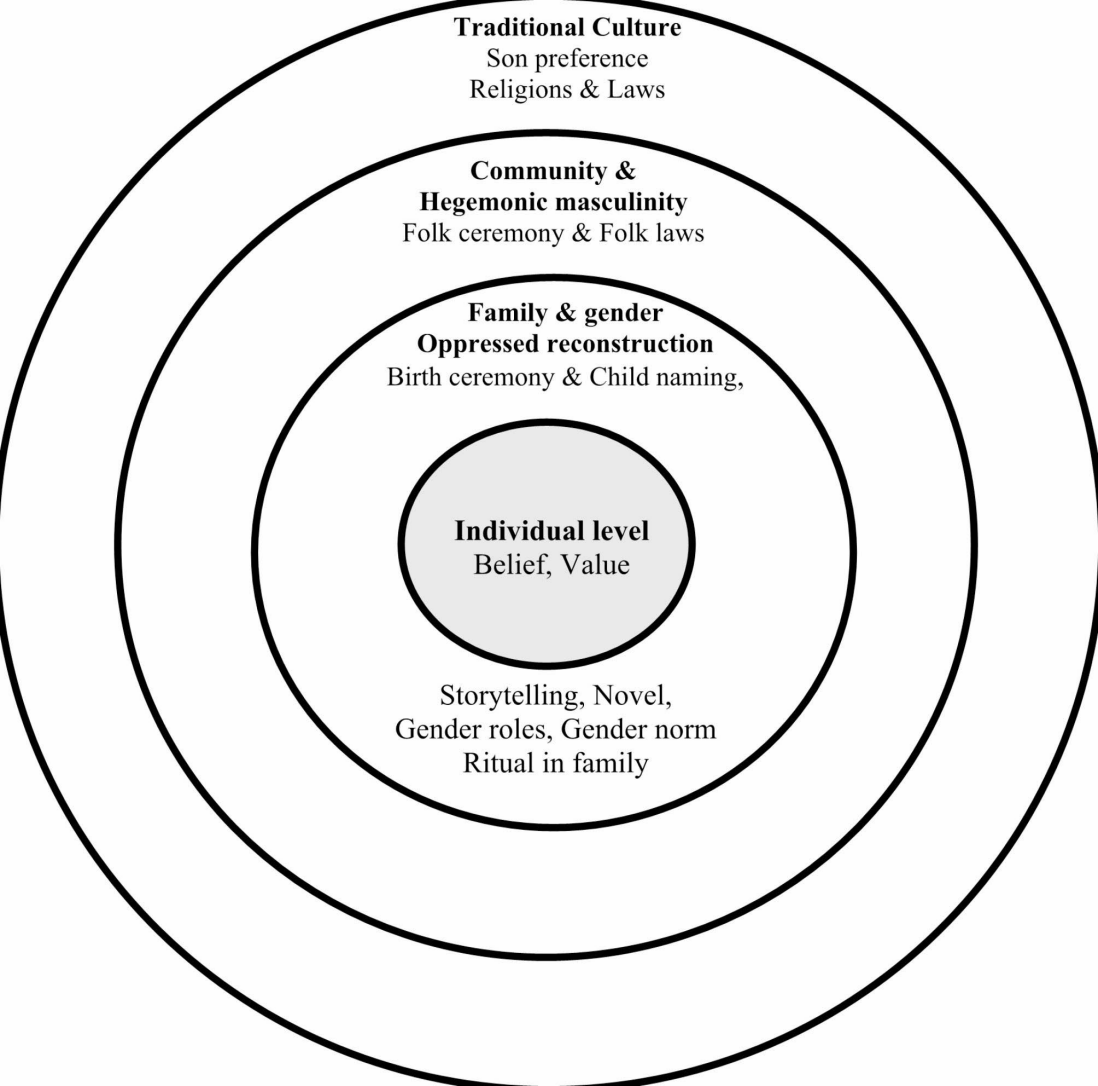
Previous days’ situation of the Akha women oppression which having a small power at the individual level, and a large power of family, community and traditional culture oppressed to them

A long path had to be traveled from the original Akha culture of oppressing women to the new generation who were moving toward equity. Oppressed Akha women have moved through four layers: individual, family, community, and traditional culture (Fig. 1).

A woman had very little power to express her rights and feelings. A woman had been treated as secondary to a man since she was a little girl through novels, religious rituals, etc. When a woman was married, she was treated as a man’s asset. Women were oppressed through several cultural practices, such as naming ceremonies, religious rituals, and individual farming performances. At the family level, wives were treated as their husband’s assets. As part of this status, when married couples experienced conflict, women were taught not to respond to their husbands. A response would result in evil happening later to her husband. However, on a positive note, once a woman was married, she was a complete member of her husband’s ancestry and could not return to her family of origin.

At the family level, mothers-in-law played a critical role in controlling, monitoring, and guiding their daughters-in-law. Mothers-in-law were external family but became part of a family after marriage. Then, a new chain began after a new daughter-in-law matured as a family member. Housework and farming were other contributors to the oppression of Akha women. Women in a family had to work hard to support all family members. When small girls or boys were born into a family, they were treated and cared for differently through novels, stories, and daily training. The different gender roles and their importance were inserted into daily practices and religious rituals.

The Akha community is surrounded by rich cultural and traditional patterns. All patterns have led to the creation of individuals’ beliefs and roles under different genders. Women have handled all the family work, including the cooking, laundry, childcare, elder care, and housework. It is widely accepted in the Akha society that all the housework, caring independent people in the family and cooking are the responsible of women. Working these jobs men are not accepted and are being looked at as a weak person. However, males, especially husbands, have played a major role in discussions with their neighbors in the morning and playing sports in the evening. Hanging up the laundry after the sunset was a major female responsibility. Men did not do this job because the women’s underwear was despicable to them.

In the context of outside villages, especially given the power of their culture and modernization, Akha women were greatly oppressed. The position of Akha women remained at the small central point of the factor circle.

**Fig. 2:**
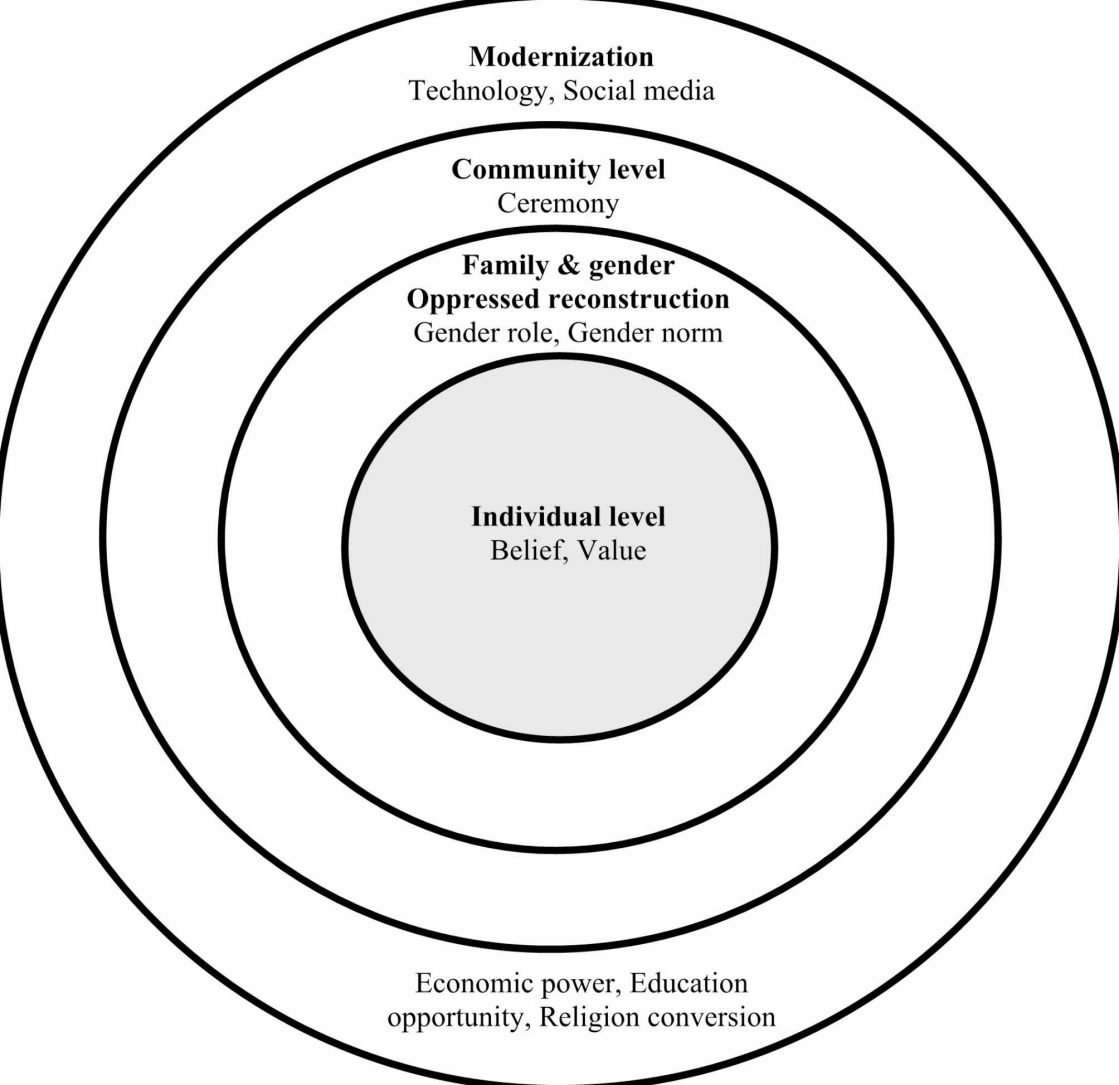
Current days on having a stronger individual power to reduce the oppression from family, and community by increasing the modernization

Today, as cultures mix, people have greater access to education, economic power, and interactions with others through different online platforms. Therefore, the circle representing Akha women, formerly the little circle in the center, is enlarging. In the context of the cultural combinations of global modernization—combined with the power of the other circles representing the family, community, and outside villages—Akha women are gaining the power to negotiate against their oppression. The current situation supports Akha women in improving their power, while the roles of Akha men, including in other sectors, were decreasing as they accept new modern cultures (Fig. 2).

## Discussion

The Akha people in Thailand have poor status and low educational attainment, and they work in the agricultural sector. Akha women had their roles and rights oppressed through many patterns, such as their daily lives, religious rituals, a culture that favors sons, stories in novels and cradle songs, naming ceremonies, and women’s work performances. Unorthodox patterns relieving women’s oppression among the Akha people were also detected through religious conversion, the impact of education, exposure to people outside the villages, and social and economic roles. The combination of cultures and globalization was a key factor in gender inequity in Akha society by balancing oppression with resistance at the individual, family, and community levels.

Akha women are oppressed through their daily lives and performance, especially in farming. Akha women must work hard in their homes to maintain the needs of all the people in their families, including cleaning the living environment. Moreover, women who have many weeds on their farms and no vegetable growth for their families are blamed for their poor performance. Women oppressed in their daily lives and performance, through their traditional cultures, have frequently been reported in developing countries, such as Zimbabwe [21] and Nigeria [22]. Neuenfeldt [23] reported that several countries, including Korea, had women oppressed throughout their daily lives. Therefore, oppressed women were more present in developing countries with traditional cultures, while many modernized countries also detected oppressed women.

In Akha culture, particularly under the traditional religion, most people prefer to have a son or at least to have a boy in their family. Son or boy domination in Akha society is taught through novels, stories, cradle songs, and naming ceremonies. Novels and cradle songs demonstrate the importance of the male role in Akha society and teach females to be subservient to their prospective husbands. Moreover, a boy’s name is inherited from his father and acts as his father’s footprint. However, girls are ignored, resulting in the belief that a girl is her husband’s future asset. A study in Saudi Arabia also found that women were oppressed by religion and culture [24]. Klingorova et al. [25] reported that religion was the major cause of gender inequality worldwide. A study conducted in Africa reported that the majority of oppressed women were oppressed through religious practices [26]. Neuenfeldt [23] also reported that naming ceremonies facilitated female oppression in several countries and cultures. Nugraheni [27] reported that women are oppressed by men through novels and cradle songs. Akha women have been oppressed in several ways, including religious and daily cultural practices, such as naming ceremonies and stories.

Several factors have contributed to improving conditions for the Akha-oppressed women. These include religious conversion to Christianity, education, exposure to people outside the village, and the impact of external social norms and economic roles. Derichs et al. [28] reported that many Asian families converted to Christianity to reduce women’s oppression and the cultural favoring of sons. From the Chinese culture, the hill tribe people in Thailand adopted the cultural favoring of sons. The basic belief is that having a boy could lead a family to have a good life after death, capable of meeting their family members who passed away.

Education is another contributing factor to gender equality in Akha society. With a high educational level, Akha women can be employed in good positions and make sufficient income to support their families. Once they have an income, women have the power to make decisions regarding purchasing, which can lead to reduced oppression from males. This finding was clear in Akha society, especially in younger families, under the impact of socioeconomic factors. The laws and regulations of Thailand guarantee equal education and work for all people. No specific organizational or traditional culture can prefer any gender for promotion. Therefore, Akha women, especially younger women, have a good opportunity to relieve their cultural suffering from women’s oppression if they can work outside their village. Doing so exposes them to people and allows them to learn the new culture of gender equity. However, only those who hold Thai identification cards are allowed to attend universities. Compared with previous days, currently, Akha women have a better education particularly those ages 30 years and below [14]. However, those people aged 30 years and over have limited in attending Thai educational system.

Through exposure to people outside the village, Akha women learned how they should behave in their daily lives. Outside the frame of their traditional cultures, Akha women freely worked and interacted with people from different backgrounds. After living for a time outside the village, Akha women could adjust how they managed their traditional practices and culture, especially those under which they were oppressed. While many Akha women had the opportunity to work outside their villages, Akha society had an increasing sense of accepting women’s role in working a good job and earning much money to support their families. As a result, the pressure to oppress them was also relieved. Because they had the power to expose people outside their village and because they were exposed to modern culture, educated, and had good opportunities to work and earn money, Akha women, especially younger Akha women, were able to be relieved of their suffering under their traditional culture of female oppression.

This study had a few limitations. First, participants aged less than 25 were not recruited into the study, and some experiences or personal ideas about women’s oppression were presented. The participants were recruited from six villages located along the border of Thailand and Myanmar, which are the original Akha villages in Thailand. This selection might impact the generalizability of the findings to the entire Akha population. There might be differences between those who live in villages located closer to cities or urban areas. More than half of the participants were Buddhist or animist, which might impact the representation of the Akha in Thailand, but it offered the best scenarios for the study objective.

## Conclusion

Akha women have been suppressed in many patterns through their daily lives ranging from religious rituals, Akha novels, naming ceremonies, and work performances. Today, Akha women are exerting their right to have equal gender roles with the support of many factors, such as religious conversion, which is a common and effective practice. Moreover, the impacts of having a higher educational level for women, exposing them to people outside their village, and modern social and economic roles are acting to balance gender roles in Akha society. With the movement of traditional cultures to oppress women’s roles and the unorthodox changes supported by modernization cultures and socioeconomics, Akha women are increasing their opportunity through the four layers–i.e., individual, family, community, and external culture and modernization, to balance their roles with Akha men, which could lead to a better stage of Akha women’s lives in the future. Implementing a specific project designed to empower women and encourage family and community roles could be a good choice to reduce women’s oppressed in Akha society. Providing an opportunity for Akha women in attending a school could improve their skills to handle the impacts of women oppressed by the external culture and modernization.

### Electronic supplementary material

Below is the link to the electronic supplementary material.

Supplementary Material 1

Supplementary Material 2

## Declarations

### Available of data and materials

Supported data for the study findings are available in supplement files.

## Abbreviations

ID: Identification card
SDG: Sustainable Development Goal
UN: United States
