# Supplementary material for: Women oppressed in the daily lives and cultural practices of the akha people, Thailand: how can the situation change?
Source: BMC Psychol. 2023 Oct 6;11:311. doi: 10.1186/s40359-023-01361-6 (PMC10557199; doi:10.1186/s40359-023-01361-6)
Supplement: Supplementary file 1 — Supplementary Material 1 [file 40359_2023_1361_MOESM1_ESM.docx]

**Focus group discussion guideline**

**Women Oppressed in the Daily Lives and Cultural Practices of the Akha people, Thailand**

Women’s oppression through Akha rituals and ceremonies

1.     What are the important rituals and ceremonies of Akha and how is it performed?

2.     How do Akha rituals and ceremonies perform differently between boys and girls?

3.     What is Akha’s love and partner selection?

4.     What is Akha’s belief about pregnancy and family planning?

5.     What is Akha’s belief about infertility women?

Gender roles and norms

6.     Let’s talk a little bit about back when you were young. What is the preferred parenting style of Akha?

7.     How does Akha nurture the children?

8.     How about the gender preference between son and daughter in the Akha family?

9.     How about Akha’s kinship system?

10.  What is the social expectation of men and women's role?

11.  What is the characteristic of good women and men in Akha perspective?

Existing forms and patterns of oppression

12.  What do you call good/bad women in Akha words or metaphors?

13.  What is the difference between a boy and girl’s education opportunity?

14.  What is the difference between man and women’s political opportunity?

15.  What is the difference between man and women’ employment opportunities?

16.  How about domestic violence in the Akha family?

17.  How does Akha deal with domestic violence?

The dynamics of traditional cultures and norms under modernization.

18.  How is Akha parenting style different from the past?

19.  How are Akha men and women's roles different from the past?

20.  How is the Akha religion and ceremony different from the past?

21.  How does Ahka women deal with gender discrimination?

22.  How does information technology impact Akha's way of life?
